# HIV-Dementia Scale as a screening tool for the detection of subcortical cognitive deficits: validation of the Italian version

**DOI:** 10.1007/s00415-021-10592-9

**Published:** 2021-05-15

**Authors:** C. Montanucci, E. Chipi, N. Salvadori, R. Rinaldi, P. Eusebi, L. Parnetti

**Affiliations:** Center for Memory Disturbances, Lab of Clinical Neurochemistry, Section of Neurology, Department of Medicine and Surgery, University of Perugia, Piazzale Gambuli 1, 06132 Perugia, Italy

**Keywords:** HIV-Dementia Scale, Subcortical cognitive impairment, Screening tools, Cognitive profile

## Abstract

Mini-Mental State Examination (MMSE) lacks of sensitivity in detecting cognitive deficits associated with subcortical damage. The HIV-Dementia Scale (HDS), a screening tool originally created for detecting cognitive impairment due to subcortical damage in HIV + patients, has proved to be useful in other neurological diseases. Until now, an Italian version of the HDS is not available. We aimed at: (1) validating the HDS Italian version (HDS-IT) in a cohort of cognitively healthy subjects (CN); (2) exploring the suitability of HDS-IT in detecting cognitive impairment due to subcortical damage (*sc*CI). The psychometric properties of the HDS-IT were assessed in 180 CN (mean age 67.6 ± 8.3, range 41–84) with regard to item-total correlation, test–retest reliability and convergent validity with MMSE. Item-total correlations ranged 0.44–0.72. Test–retest reliability was 0.70 (*p* < 0.001). The HDS-IT scores were positively associated with MMSE score (rS = 0.49, *p* < 0.001). Then, both the HDS-IT and the MMSE were administered to 44 *sc*CI subjects (mean age 64.9 ± 10.6, range 41–84). Mean HDS-IT total score was close to the original version and significantly lower in the *sc*CI group compared to CN (8.6 ± 3.6 vs. 12.6 ± 2.5, *p* < 0.001). ROC analysis yielded an optimal cutoff value of 11, with sensitivity of 0.70 and specificity of 0.82. Patients showed poorer scores on HDS-IT compared to CN (12.6 ± 2.5 vs. 8.6 ± 3.6, *p* < 0.001). Our results support the use of HDS-IT as a screening tool suitable for detecting cognitive deficits with prevalent subcortical pattern, being complementary to MMSE in clinical practice.

**Supplementary Information:**

The online version contains supplementary material available at 10.1007/s00415-021-10592-9.

## Introduction

Early detection of cognitive impairment in the ageing population represents an important issue. It is known that mild cognitive impairment (MCI) in adult patients is a frequent and heterogeneous condition that may be related to different underlying causes, especially neurodegenerative or cerebrovascular diseases [1, 2]. Neurological disorders affecting the central nervous system are associated with a wide spectrum of clinical manifestations, often accompanied by presence of such cognitive dysfunctions, and in some cases, dementia. To optimise the diagnostic workup, subjects should undergo a screening assessment for the characterisation of global cognitive profile, followed by an extensive assessment if cognitive deficits are detected [3]. Accordingly, an ideal screening tool should be relatively simple to administered, not time consuming and sensitive enough to allow the identification of patients deserving further, in-depth neuropsychological assessment [4]. In particular, screening tests for the assessment of global cognitive functioning should be able to highlight cognitive profiles with prevalent cortical (i.e. deficits in declarative memory, language, praxis and visuospatial abilities) vs. subcortical (i.e. deficits in attention and arousal, memory retrieval, speed of information processing, motivation and mood) pattern of cognitive impairment, so that clinicians may be better oriented in their examination. [5–7]. Anatomically, the cortical pattern is related to diseases involving primarily, but not exclusively, the association cortex of the cerebral hemispheres and the medial temporal lobes, and are typically characterised by aphasia, amnesia, agnosia, acalculia, and apraxia. The subcortical pattern occurs in disorders with predominant involvement of basal ganglia, thalamus, and structures of the brainstem, and is typically characterised by psychomotor slowing, memory impairment, affective and emotional disorders, and difficulties with strategy formation and problem solving [8]. However, though the historic cortical vs. subcortical dichotomy may be useful to identify preeminent neuropsychological profiles in clinical practice [9], the existence of “true” cortical and subcortical disorders is controversial from a functional/neuroanatomical perspective [10].

In clinical practice, the most popular screening tool for assessing global functioning is the Mini-Mental State Examination (MMSE) [11]. MMSE is composed by several items, most of them requiring the integrity of higher cortical functions (memory, language, orientation and visuo-constructive praxis). However, MMSE lacks of items assessing executive functions; so far, its sensitivity in detecting subcortical patterns of cognitive impairment is low [7, 12–14]. Several studies reported that the Montreal Cognitive Assessment (MoCA), another well-known screening test, is superior to MMSE for the early detection of cognitive impairment in ageing population [14, 15], due to its item composition. In fact, the MoCA is more suitable than MMSE in assessing visuospatial and executive functions, representing a more challenging task to be used in clinical practice [3]. On the other hand, a brief bedside screening tool as the Frontal Assessment Battery (FAB) is ideal for the assessment of executive functions, despite, it cannot replace measures of global cognition as the MMSE. Studies that investigated the utility of FAB for differential diagnosis among different dementias gave mixed results, showing more suitability of specific FAB sub-items than the FAB total score in distinguishing cortical dementias, as Alzheimer’s disease and fronto-temporal lobar dementia, and subcortical vascular cognitive impairment [16–18]. However, none of the abovementioned screening tools is adequate for assessing reaction times and speed processing, because both of them lack of time-dependent items.

Actually, subcortical cognitive impairment (*sc*CI) is mainly related to damage in specific subcortical brain regions (i.e. thalamus, basal ganglia, midbrain), but it may also be a consequence of disruption in white matter connection fibres (white matter lesions, WMLs). Accordingly, pictures of WMLs, that may disrupt cortico-cortical intra- and inter-hemispheric, as well as cortico-subcortical connections, may cause this kind of cognitive impairment [19]. *sc*CI represents a clinical feature of many neurological diseases, such as subcortical ischaemic vascular disease (SIVD), normal pressure hydrocephalus (NPH), multiple sclerosis (MS), Huntington’s disease (HD), Parkinson’s disease (PD), and progressive supranuclear palsy (PSP) [20]. Nowadays, it is known that, in clinical practice, an accurate neuropsychological assessment may allow to early detect clinical manifestations of these neurological disease prior to the dementia phase. Among the available tools for *sc*CI detection, none is suitable to be applied as screening measure in clinical practice [21]. So far, a sensitive tool for revealing features of subcortical cognitive impairment is strongly recommended. The HIV-Dementia Scale (HDS) is a brief tool originally developed to assess subcortical deficits in individuals affected by HIV infection [22]. Since HDS proved to be useful in detecting *sc*CI in HIV + patients, its suitability for detecting cognitive impairment in other neurological diseases with subcortical damage has been assessed, giving significant results in NPH and SIVD [21].

Later on, the International version of the HIV-Dementia Scale (I-HDS) [12] has been validated as a cross-cultural screening test to use for detection of HIV dementia within the worldwide community. Until now, normative data for the Italian population are lacking.

The objectives of this study are: (1) to carry out the validation of the HDS Italian version in a cohort of cognitively healthy elderly subjects (CN), and (2) to explore its sensitivity and specificity in detecting subcortical cognitive deficits in a clinical sample of subjects with neurological diseases associated with subcortical damage (*sc*CI).

## Materials and methods

### Participants and assessment procedure

We enrolled 180 CN recruited among relatives of patients attending our Memory Clinic or as volunteers after advertisement. The participants’ inclusion criteria included: (i) age between 40 and 85, (ii) good physical and mental health, (iii) no concomitant uncontrolled medical diseases, (iv) Mini-Mental State Examination raw score ≥ 24, and (v) no dementia. Patients were classified as CN by means of extensive neuropsychological evaluation (see section below) assessing multiple cognitive domains. Scores within normal range in all cognitive domains led us to define a patient as CN. A subgroup of 27 subjects repeated the HDS-IT after a mean test–retest interval of 3–10 months (median 7).

We also enrolled 44 consecutive patients attending to our Memory Clinic for neurological disorders with subcortical features: 13 with multiple sclerosis (MS), 16 with subcortical ischaemic vascular disease (SIVD), 9 with normal pressure hydrocephalus (NPH) and 6 with HIV+ infection (HIV+). For patients with diagnosis of MS, we adopted the radiological criteria of minimum 4–9 white matter lesions [23]. For SIVD patients, we adopted the radiological criteria of score 2–3 in the Fazekas scale [24]. Patients with NPH were included on the basis of clinico-radiological diagnosis. Patients with HIV+ were included on the basis of serological diagnosis.

All of them showed subcortical cognitive impairment (*sc*CI). *Sc*CI was defined as a score ≥ 1.5 SD below the adjusted-mean in one or more cognitive domains, evaluated through an extensive neuropsychological battery, in patients with neurological disorders associated with subcortical damage. A clinical condition of dementia was excluded for all patients.

### Neuropsychological testing

All subjects underwent the following neuropsychological battery: the MMSE [25] for the assessment of global cognitive functioning; the Rey Auditory Verbal Learning Test [26] for the evaluation of verbal learning and memory; the digit span forward and backward [27] for the evaluation of verbal short-term memory and working memory; the Trail Making Test-part A and B [28] for the evaluation of visuospatial selective and divided attention and mental shifting; the copy of drawings and copy of drawings with landmarks [26] and the Clock Drawing Test [29] for the evaluation of visuo-constructive praxis; the Raven’s coloured progressive matrices ‘47 [30] for the evaluation of abstract logical reasoning; the phonemic fluency [31] and category fluency [32] for the evaluation of language. Clinical staging was assessed by means of the Clinical Dementia Rating Scale (CDR) [33].

### The original version of HIV-Dementia Scale

The HDS original version consists of four subtests. Item 1—attention (max score = 4): modified from anti-saccadic error task [34]. The patient is asked to look the examiner’s nose and then to focus on examiner’s moving index finger, repeating the task with alternating hands. When the patient is comfortable, looking at the finger that moves, the examiner ask him/her to look at the not moving index finger. This task is practised until the patient becomes familiar with the procedure. Then the patient is asked to perform 20 serial anti-saccades. An error is marked when the patient looks towards the moving finger. Item 2—psychomotor speed (max score = 6): patient is asked to write the entire alphabet. If the patient is unable to perform it correctly, the examiner asks him/her to write the numbers from 1 to 26 and the time taken is recorded. The time taken to complete this task is converted into a numerical value from 0 to 6. Items 3—memory recall (max score = 4): the patient is asked to repeat and remember four words. The four words to be recorded in the HDS-IT memory subtest correspond to the translation of the original version (“dog”, “hat”, “green” and “peach”) [21]. Item 4—construction speed (max score = 2): the patient is asked to the draw of a copy cube. Primarily the examiner explains the figure copy, the time needed to copy is recorded and converted into a numerical score from 0 to 2. The maximum HDS score is 16.

### Development of the Italian version

The HDS-IT was developed using forward–backward translation. Two researchers separately translated the English version into Italian, and then compared the two translations. The resulting draft was translated back into English by a native independent English speaker fluent in Italian language who did not know the original version of the scale. The Italian version was compared with the original English version, any discrepancy was discussed and a final version was adopted after reaching the full agreement. This final Italian version is reported in Supplementary file. As with the original English version, in HDS-IT the item of psychomotor speed consists of writing the numbers from 1 to 21 (instead of 1–26 as in the original version), due to Italian alphabet, composed of 21 letters rather than 26 as the English one.

As for the original version, the maximum score is 16.

To avoid interference with the recall of words (for instance, ‘CAPPELLO’ may interfere with the RAVLT list), we suggest that the administration of the test should be done at least 15 min before the verbal memory test.

### Statistical analysis

The data were analysed using R version 3.5. Descriptive statistics were calculated. Student *T* test was applied to test significance of differences of continuous variables between *sc*CI and CN. Mann–Whitney *U* test was used whenever appropriate. Gender difference between the groups was assessed via Chi-Square Test. Test–retest variability was calculated with Spearman correlation coefficient. Receiver-operating characteristics (ROC) curve analysis was carried out for evaluating accuracy of HDS in discriminating *sc*CI from controls. The optimal cutoff value was determined according to Youden Index. AUC, sensitivity and specificity were provided along with their 95% CI according with the selected cutoff. In all the analyses, two-sided *p* values < 0.05 were considered as statistically significant.

## Results

Demographic characteristics of participants are reported in Table 1. CN and *sc*CI did not differ in the distribution of age, gender and education. CN subjects showed lower MMSE mean scores compared to *sc*CI (26.2 ± 2.8 vs. 28.3 ± 1.3, *p* < 0.001).

**Table 1:** Demographical and clinical features of the study cohort

	All	CN	*sc*CI	*p* value
N	224	180	44	–
Gender, male/female	97/127	76/104	21/23	0.624
Age (years ± SD)	67 ± 8.8	67.5 ± 8.3	64.9 ± 10.6	0.135
Education (years ± SD)	11.3 ± 4.3	11.3 ± 4.1	11.1 ± 4.8	0.726
MMSE (score ± SD)	27.9 ± 1.93	28.3 ± 1.3	26.2 ± 2.8	< 0.001

Age, education and Mini-Mental State Examination (MMSE) scores are reported as mean ± standard deviation (SD)

*CN* subjects without subcortical cognitive impairment, *scCI* subjects with subcortical cognitive impairment, *MMSE* Mini-Mental State Examination, *SD* standard deviation

### Validation of the HDS Italian version

The HDS-IT total score was negatively associated with age (rS = − 0.18, *p* = 0.008), while it was positively associated with education (rS = 0.39, *p* < 0.001). No associations were found with gender (*p* = 0.571). HDS-IT and MMSE total scores were positively associated (rS = 0.49, *p* < 0.001).

Corrected item-total correlations ranged between 0.44 and 0.72. Moreover, the average of inter-item correlation was higher than 0.17. Test–retest reliability was assessed in 27 subjects, yielding a score of rS = 0.70 (*p* < 0.001). The mean duration between visits was 3–10 months (median: 7).

### Exploring HDS-IT suitability in detecting subcortical cognitive deficits

Mean HDS-IT total score was close to the original version in both groups and significantly lower in the *sc*CI compared to CN group (12.6 ± 2.5 vs. 8.6 ± 3.6, *p* < 0.001). Performing a sub-analysis comparing single items in the two groups, we found significant differences for item 2 (5.2 ± 1.4 vs. 2.7 ± 2.5, *p* < 0.001), and a trend toward significance for item 3 (3.0 ± 0.9 vs. 2.1 ± 1.2, *p* = 0.004). All complete results are shown in Table 2.

**Table 2:** Differences in HDS-IT scores within CN and *sc*CI groups

	CN	*sc*CI	*p* value
HDS-IT total score	12.6 ± 2.5	8.6 ± 3.6	< 0.001
HDS-IT item 1 (anti-saccadic eye movements)	3.3 ± 1.1	3.1 ± 1.3	0.479
HDS-IT item 2 (numerical series)	5.2 ± 1.4	2.7 ± 2.5	< 0.001
HDS-IT item 3 (memory task)	3.0 ± 0.9	2.1 ± 1.2	0.004
HDS-IT item 4 (cube copy)	1.0 ± 0.9	0.5 ± 0.8	0.013

Values are reported as mean scores ± standard deviations

*CN* subjects without subcortical cognitive impairment, *scCI* subjects with subcortical cognitive impairment

**Fig. 1:**
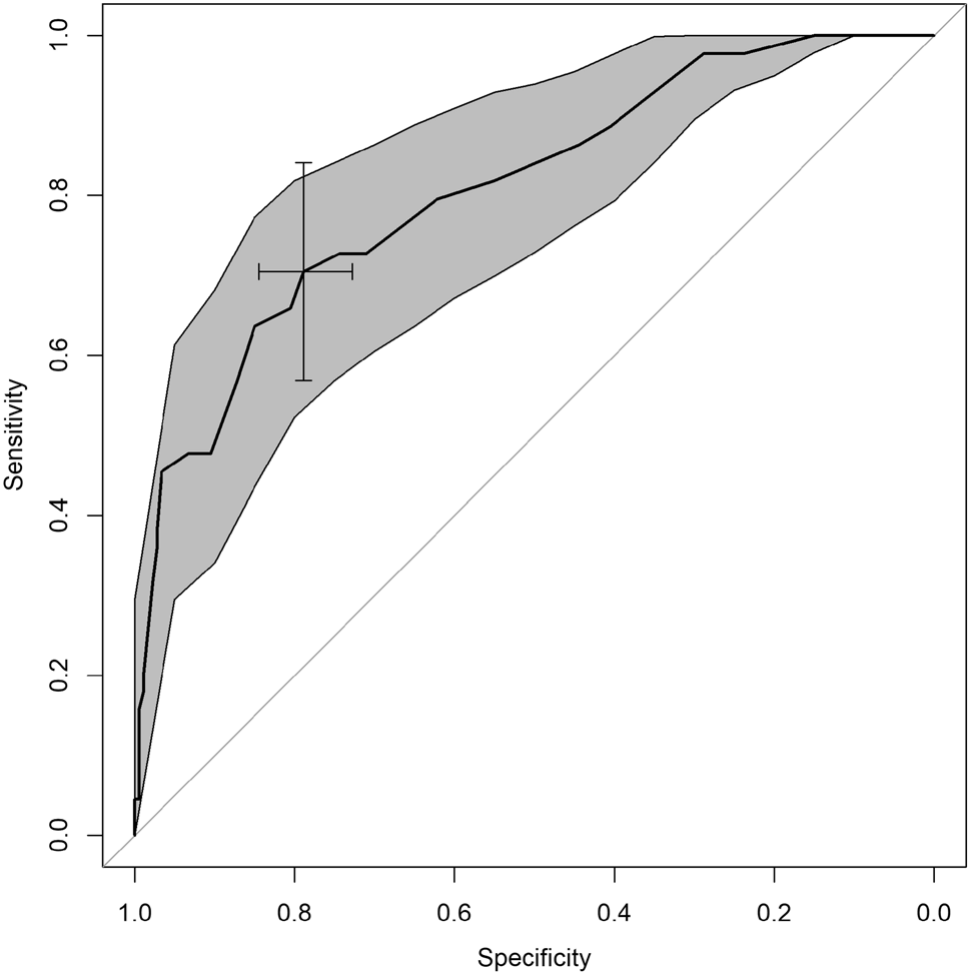
ROC curve for HDS-IT values to determine the optimal cutoff score to identify *scCI*

To determine the optimal cutoff score for identifying *sc*CI versus CN with the HDS-IT, a ROC curve was constructed (Fig. 1) and was adjusted for age. The ROC curve (AUC = 0.80, 95% CI 0.73–0.88) yielded an optimal cutoff value for an HDS-IT score of ≤ 11. Based on this cutoff value, the sensitivity was 0.70 (95% CI 0.48–0.84) and the specificity 0.82 (95% CI 0.65–0.97). A score of ≤ 11 was also able to discriminate those *sc*CI with MMSE ≥ 24 (37/44) vs. CN, with a sensitivity of 0.65 (95% CI 0.49–0.81) and a specificity of 0.79 (95% CI 0.73–0.85).

## Discussion

The purposes of this study were to validate the Italian version of the HDS (HDS-IT) in a cohort of cognitively healthy elderly subjects and to explore its suitability as a sensitive screening tool for detecting subcortical cognitive impairment in subjects with neurological diseases associated with subcortical damage (*sc*CI).

With respect to the first aim (validation of the HDS-IT in a cohort of cognitively healthy volunteers), our results displayed that the HDS-IT revealed good psychometric properties as well as the original version, shown by the criterion validity and test–retest reliability. Regarding the criterion validity, we found a trend in convergent validity between HDS and MMSE (i.e. the better the MMSE score, the better the HDS score). However, these measures did not overlap, because of their complementarity due to different items composition. About test–retest reliability, we found a robust test–retest correlation (rS = 0.70) with a mean interval of 3–10 months. Our result was in line with a previous study that found good performance at 3–9 weeks interval [22] and at 4 months [35]. The use of a wider time interval in our study may represent an advantage to control for possible learning or practice effect, defined as “the capability of an individual to learn and adjust” after repeated administration of a task [36]. This represents a critical issue in clinical practice, since this can affect the test–retest reliability of a task, particularly in cognitively healthy subjects [37, 38]. Furthermore, we found that the HDS-IT was inversely associated with age and positively associated with education, whereas it was independent from gender. Our findings are in line with that observed for other screening tests, so a correction for age and education should be considered in future studies, for a better interpretation of the raw scores obtained at the HDS-IT.

With regard to the second aim (i.e. the behaviour of the HDS-IT in a clinical sample of scCI patients compared to healthy control), we found that patients with *sc*CI showed poorer scores on the HDS-IT compared to cognitively healthy individuals, even in those with MMSE < 28. This observation further supports the sensitivity of the HDS-IT in detecting cognitive deficits with prevalent subcortical pattern.In particular, those *sc*CI patients who displayed normal scores on MMSE frequently displayed low scores on the HDS-IT. A previous study found significant correlations of the HDS scores with neuropsychological measures exploring attention/working memory, processing speed and executive functions, supporting the usefulness of this test for detection of subcortical cognitive deficits [39]. In our study, the capability of HDS-IT in detecting subcortical cognitive impairment was accomplished by comparing HDS-IT performance between patients with *sc*CI and CN. Overall, our results support the use of HDS as a screening tool for detecting subcortical cognitive deficits, being complementary to MMSE in clinical practice.

Our study has some limitations. While all patients in the *sc*CI group underwent a neuroimaging acquisition, this was not provided for some individuals in the control group. Therefore, the actual vascular load (i.e. white matter hyperintensities and subcortical damage) might have been underestimated in the control group. Moreover, the *sc*CI group was heterogeneous in terms of diagnosis, although all of the patients shared a subcortical pathophysiology underlying their neurological disease. Only 6 patients with HIV infection have been included, diagnosed on the basis of clinical and laboratory data, while no brain imaging data were available. Therefore, in the present study, the suitability of the Italian version of the HDS in the original test’s target population has not been replicated.

Further studies have to be performed to validate the HDS in other diseases causing *sc*CI, such as Parkinson’s disease or Huntington’s disease, as recommended previously [21]. Furthermore, future studies can be useful to explore the performance at HDS-IT also in cortical-type neurodegenerative diseases (i.e. Alzheimer’s disease), where a subcortical damage may be involved in the pathogenesis and accompany the neurodegenerative processes, though at a lower level of magnitude.

In conclusion, our results suggest that the HDS-IT is able to detect subcortical deficits in a population of patients with subcortical neurological disorders (i.e. SIVD, NPH and MS and HIV+). The HDS-IT showed good psychometric properties, so it may represent a suitable screening tool to be used in clinical practice, being complementary to MMSE.

## Supplementary Information

Below is the link to the electronic supplementary material.

Supplementary file1 (DOCX 23 KB)

## Data Availability

Data and material are available upon reasonable request.
