# Supplementary material for: HIV-Dementia Scale as a screening tool for the detection of subcortical cognitive deficits: validation of the Italian version
Source: J Neurol. 2021 May 15;268(12):4789–95. doi: 10.1007/s00415-021-10592-9 (PMC8563637; doi:10.1007/s00415-021-10592-9)
Supplement: Supplementary file 1 — Supplementary file1 (DOCX 23 KB) [file 415_2021_10592_MOESM1_ESM.docx]

HIV – *Dementia Scale Versione Italiana* (HDS-IT)

1. **REGISTRAZIONE:**

“*Questa è una prova di memoria. Le leggerò un elenco di parole, ascolti attentamente. Quando avrò finito, mi dica tutte le parole che riesce a ricordare. Non importa l’ordine in cui le dice. È pronto/a? Le parole sono: CANE, CAPPELLO, VERDE, PESCA*”.

Leggere le parole impiegando un secondo per ciascuna.

*“Le leggerò di nuovo lo stesso elenco. Quando avrò finito mi dica tutte le parole che riesce a ricordare: CANE, CAPPELLO, VERDE, PESCA”.*

*“Tenga in mente le parole, tra qualche minuto le chiederò di ripeterle”.*

1. **ATTENZIONE:**

L’esaminatore alza il dito indice destro all’altezza degli occhi del paziente ed invitarlo a fissarlo. Successivamente, alza l’indice sinistro, posizionato ad una distanza dall’altro pari alla larghezza delle sue spalle ed invita il paziente a fissarlo.

*“Le chiedo di fissare il mio dito (indice destro). Adesso invece guardi qui (indice sinistro)”.*

Assicurarsi che il paziente abbia appreso il compito. L’esaminatore chiede poi di fissare nuovamente l’indice destro che rimane fermo, e contemporaneamente muove in avanti e indietro l’indice sinistro per 5 volte.

*“Ora guardi di nuovo qui (indice destro)”.*

Subito dopo, ripete la stessa operazione chiedendo di fissare l’indice sinistro (che rimane fermo) mentre con l’indice destro effettua 5 movimenti.

*“Ora guardi di nuovo qui (indice sinistro)”.*

Numero di errori: ______

≤ 3 errori = 4; 4 errori = 3; 5 errori = 2; 6 errori = 1; > 6 errori = 0

PUNTEGGIO OTTENUTO: / 4

1. **VELOCITÀ PSICOMOTORIA:**

*“Le chiedo di scrivere tutti i numeri in progressione da 1 fino a 21. Le prenderò il tempo, perciò cerchi di farlo più rapidamente possibile”.*

Registrare il tempo di esecuzione della prova.

Tempo impiegato: ______ sec.

≤ 21 s = 6; 21-24 sec = 5; 24,1-27 = 4; 27,1-30 s = 3; 30,1- 33 s = 2; 33,1-36 s = 1; > 36 s = 0

PUNTEGGIO OTTENUTO: / 6

1. **RICHIAMO:**

*“Prima le ho letto un elenco di parole che le avevo chiesto di tenere in mente. Mi dica tutte le parole che riesce a ricordare.”*

Se le parole non vengono rievocate spontaneamente, dare un aiuto di tipo semantico, cioè: dire “animale” per far ricordare la parola “cane”, “qualcosa che si indossa” per ricordare “cappello”, “colore” per ricordare “verde”, “frutto” per ricordare “pesca”.

Assegnare 1 punto per ogni parola ricordata spontaneamente, 0.5 per ogni parola ricordata con l’aiuto.

PUNTEGGIO OTTENUTO: / 4

1. **COSTRUZIONE:**

*“Le chiedo di ricopiare questa figura cercando di essere più preciso e rapido possibile. Le prenderò il tempo”.*

Registrare il tempo di esecuzione della prova.

Tempo impiegato: ______ sec.

<25s = 2; 25-35s = 1; >35 = 0; cubo scorretto = 0

Assegnare punteggio 0 se è presente almeno una delle seguenti: copia completata in > 35 secondi o il disegno è scorretto*

*La copia non è tridimensionale; le linee non sono parallele e/o non si incontrano a formare gli angoli

PUNTEGGIO OTTENUTO: / 2

PUNTEGGIO HDS-IT: /16
